# Hemorrhage distribution, imaging-defined post-hemorrhagic ventricular dilatation, and clinical outcomes in neonates with intraventricular and cerebral parenchymal hemorrhage: a single-center retrospective cohort study

**DOI:** 10.3389/fped.2026.1965213

**Published:** 2026-10-22

**Authors:** Xuehua Zhang, Youchun Cai, Saifeng Huang, Huiqing Shi, Lingting Chen, Shuihua Wu, Yonghua Xiang

**Affiliations:** 1 Fujian Maternity and Child Health Hospital, College of Clinical Medicine for Obstetrics & Gynecology and Pediatrics, Fujian Medical University, Fuzhou, China; 2 Department of Ultrasound, Fujian Children’s Hospital (Fujian Branch of Shanghai Children’s Medical Center), College of Clinical Medicine for Obstetrics & Gynecology and Pediatrics, Fujian Medical University, Fuzhou, China; 3 The Affiliated Children’s Hospital of Xiangya School of Medicine, Central South University (Hunan Children’s Hospital), Changsha, China

**Keywords:** cranial ultrasound, intraventricular hemorrhage, magnetic resonance imaging, neonate, parenchymal hemorrhage

## Abstract

**Objective:**

Neonatal intracranial hemorrhage contributes substantially to neonatal mortality and neurodevelopmental impairment. This retrospective single-center cohort study aimed to investigate associations between hemorrhage anatomical distribution, post-haemorrhagic ventricular dilation severity, and clinical outcomes among neonates with intraventricular and/or cerebral parenchymal hemorrhage, and to identify factors associated with mortality and Neurodevelopmental abnormality.

**Methods:**

This retrospective cohort study included 109 neonates with intraventricular hemorrhage (IVH) and/or cerebral parenchymal hemorrhage (CPH) hospitalized at Hunan Children's Hospital from 2015 to 2018. Patients were classified into isolated IVH, isolated CPH, and combined IVH with parenchymal involvement groups. Clinical characteristics, systemic comorbidities, PHVD severity, treatment interventions, and follow-up outcomes were analyzed. MRI findings were additionally evaluated in infants who underwent examination. Multivariable logistic regression was performed to identify factors associated with clinical outcomes.

**Results:**

Among the 109 neonates, 44 (40.4%) had isolated IVH, 41 (37.6%) had isolated CPH, and 24 (22.0%) had combined IVH and parenchymal involvement. Imaging-defined post-hemorrhagic ventricular dilatation (PHVD) severity differed significantly among groups (*P* < 0.001), with moderate-to-severe imaging-defined PHVD occurring in 61.4%, 24.4%, and 70.8% of patients, respectively. Overall outcomes included 41 deaths (37.6%), 21 cases of Neurodevelopmental abnormality (19.3%), and 47 favorable outcomes (43.1%), with no significant difference among hemorrhage groups (*P* = 0.693). MRI provided additional identification of deep, posterior fossa, subarachnoid, and small focal hemorrhagic lesions. Lower gestational age and higher clinical severity scores were independently associated with mortality, whereas cerebrospinal fluid drainage or neurosurgical intervention was associated with lower odds of Neurodevelopmental abnormality (OR = 0.115, 95% CI: 0.022–0.595, *P* = 0.010). This observed association cannot be interpreted as causal or preventive therapeutic effect owing to inherent selection and survivorship bias in a retrospective observational study.

**Conclusion:**

Hemorrhage distribution was associated with PHVD severity but not independently with mortality or Neurodevelopmental abnormality. Ventricular involvement identifies infants at higher risk of significant hydrocephalus, while gestational maturity and systemic disease burden remain important determinants of outcome. Ultrasound and MRI provide complementary information for clinical assessment. The observed association between cerebrospinal fluid-directed drainage or neurosurgical intervention and favourable neurological outcomes should be interpreted cautiously. Given the retrospective non-randomized nature of this cohort, causal inference regarding preventive benefit of these interventions is not feasible.

## Introduction

Neonatal intracranial hemorrhage represents a major cause of brain injury in newborn infants, especially preterm neonates, and correlates with elevated risks of mortality, post-hemorrhagic ventricular dilation, Neurodevelopmental abnormality, and long-term neurodevelopmental impairment (1, 2). Vulnerability of the immature brain to hemorrhagic damage arises from key features of cerebral circulation: fragile germinal-matrix vasculature, impaired cerebral autoregulation, and susceptibility to abrupt systemic hemodynamic shifts in the perinatal period (3, 4). Although germinal-matrix-origin intraventricular hemorrhage (IVH) is the classic hemorrhagic lesion in preterm infants, neonatal intracranial hemorrhage presents diverse anatomical patterns, involving ventricles, cerebral parenchyma, thalamo-basal ganglia structures, cerebellum, and subarachnoid space (5–8).

The anatomical distribution of hemorrhage may reflect distinct pathogenic mechanisms, tissue-injury magnitude, and subsequent complication risks (9–12). Ventricle-confined hemorrhage primarily disrupts cerebrospinal-fluid circulation and predisposes to post-hemorrhagic ventricular dilation, while parenchymal hemorrhage may produce direct tissue destruction via separate neurological-injury pathways. These lesions frequently overlap clinically; parenchymal bleeding may extend into ventricles or co-occur with IVH. Consequently, conventional IVH grading alone cannot fully capture heterogeneous lesion patterns or their clinical consequences. An anatomy-based classification system may better characterize disease burden and support individualized risk stratification.

Cranial ultrasound serves as the first-line imaging modality for neonatal intracranial hemorrhage owing to its accessibility, portability, repeatability, and capacity for bedside monitoring in critically ill neonates (8, 13). Serial ultrasound enables tracking of hemorrhage evolution and ventricular enlargement without sedation or transport-related hazards (8, 14). Nevertheless, its diagnostic performance is limited by fontanelle acoustic windows, operator expertise, and lesion characteristics. Small parenchymal lesions, deep thalamo-basal-ganglia or posterior-fossa hemorrhage, and subtle subarachnoid microhemorrhages often escape detection on conventional cranial ultrasound (15, 16).

Magnetic resonance imaging (MRI) delivers superior soft-tissue contrast and provides supplementary information for deep-brain structures, small hemorrhagic foci, and concurrent white-matter abnormalities (17, 18). Susceptibility-weighted MRI sequences further improve identification of occult hemorrhagic components and comprehensive brain-injury assessment (19). Still, routine MRI is challenging for unstable critically-ill neonates because of clinical status, transport risks, and limited availability (20). Ultrasound and MRI are therefore complementary rather than interchangeable imaging tools. Real-world data describing additional diagnostic value from MRI beyond cranial ultrasound remain limited for neonatal hemorrhagic brain injury.

Post-hemorrhagic ventricular dilation is one of the most clinically significant complications after neonatal intracranial hemorrhage. It develops through multiple mechanisms: intraventricular blood-product obstruction of cerebrospinal-fluid pathways, impaired CSF resorption, and inflammatory responses triggered by blood-degradation products (21, 22). Existing literature has mainly focused on IVH among extremely preterm infants (23). Comparatively fewer studies have investigated ventricular-dilation risks across different anatomical hemorrhage subtypes. It remains unclear whether isolated IVH, isolated parenchymal hemorrhage, and mixed combined lesions differ in ventricular-dilation risk and long-term neurological outcomes. Furthermore, while gestational maturity and systemic comorbidities are known outcome contributors (24, 25), the relative contributions of clinical severity, hemorrhage features, ventricular injury, and therapeutic interventions toward mortality and neurodevelopmental impairment require further clarification.

To fill these knowledge gaps, we conducted a retrospective cohort study of neonates with IVH and/or cerebral parenchymal hemorrhage (CPH), categorizing lesions by anatomical involvement. We compared clinical profiles, imaging findings, and post-hemorrhagic ventricular-dilation severity across hemorrhage subgroups. We additionally reviewed MRI data in a subgroup of surviving infants to characterize lesions poorly visualized by ultrasound. Multivariable analyses were performed to identify clinical and imaging factors associated with mortality and Neurodevelopmental abnormality. This study aims to improve understanding of heterogeneous neonatal hemorrhagic brain injury and inform imaging surveillance and early risk-stratification strategies.

## Materials and methods

### Study design and population

This retrospective cohort study was conducted at the Department of Neonatology, Hunan Children's Hospital. We reviewed the medical records of infants admitted between January 2015 and December 2018 who were diagnosed with IVH, CPH, or both based on cranial ultrasound examination. The date of the first imaging examination confirming hemorrhage was considered the study entry point. The study included systematic review of clinical characteristics, neuroimaging findings, progression of ventricular dilation, and neurodevelopmental outcomes during follow-up.

Infants were eligible if they met the following criteria: admission within 28 days after birth; cranial ultrasound-confirmed IVH, CPH, or combined hemorrhagic lesions; and availability of at least one primary outcome measurement. Patients were excluded if they had congenital brain malformations, confirmed inherited metabolic or neurological disorders, isolated germinal matrix hemorrhage without parenchymal hemorrhage, periventricular hemorrhagic infarction, inadequate imaging quality preventing hemorrhage classification, or insufficient follow-up information for outcome assessment. The study protocol was approved by the institutional ethics committee of Hunan Children's Hospital. Because of the retrospective nature of the study, the requirement for informed consent was waived. This was a consecutive retrospective cohort of neonates. Study eligibility allowed hospital admission up to 28 days after birth. For infants transferred to our center beyond 72 postnatal hours, the earliest interpretable cranial ultrasonography performed either at the referring hospital or upon admission was used to identify intraventricular hemorrhage and/or cerebral parenchymal hemorrhage (IVH and/or CPH). Infants transferred after the first few days of life were included when early ultrasound confirmed IVH and/or CPH; those without interpretable early ultrasound records were excluded. The full study screening, exclusion, MRI availability and neurodevelopment follow-up process are summarized in Figure 1.

**Figure 1:**
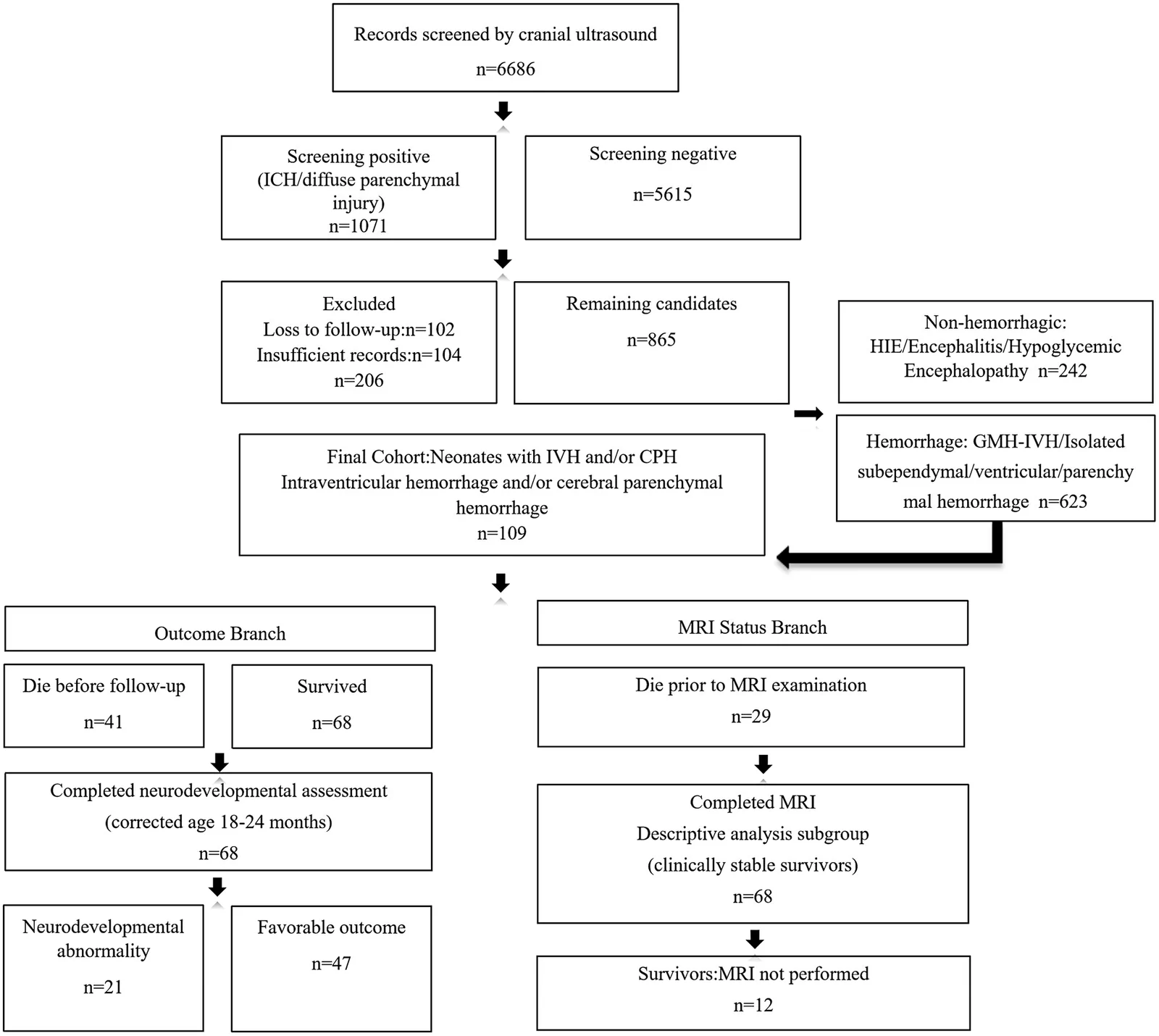
STROBE compliant participant flow diagram.

### Hemorrhage anatomical classification

Hemorrhagic lesions were classified into three mutually exclusive anatomical subgroups: isolated intraventricular hemorrhage (IVH), isolated cerebral parenchymal hemorrhage (CPH), and combined IVH and CPH.

- Isolated IVH was defined as hemorrhage confined exclusively to the ventricular system, without evidence of cerebral parenchymal hemorrhage.
- Isolated CPH was defined as hemorrhage located within the cerebral parenchyma, including thalamic-basal ganglia hemorrhage and cerebellar hemorrhage, without associated intraventricular hemorrhage.
- Combined IVH and CPH was defined as the presence of both intraventricular hemorrhage and cerebral parenchymal hemorrhage on definitive imaging.

In this study, no distinction was made between primary parenchymal hemorrhage extending into the ventricle and primary IVH with secondary parenchymal involvement. Any case showing both ventricular and parenchymal hemorrhage was classified into the combined IVH and CPH group, regardless of the presumed direction of hemorrhage spread.

Regarding specific hemorrhage subtypes:

- Isolated germinal matrix hemorrhage without parenchymal hemorrhage and periventricular hemorrhagic infarction were excluded from the study.
- Thalamic–basal ganglia hemorrhage and cerebellar hemorrhage were categorized as cerebral parenchymal hemorrhage.

### Cranial ultrasound examination and imaging-defined post-hemorrhagic ventricular dilatation (PHVD) assessment

Bedside cranial ultrasound was performed through the anterior fontanelle whenever the infant's clinical condition allowed. The initial cranial ultrasound examination was completed within 72 h after hospital admission. According to institutional clinical practice, follow-up ultrasound assessments were generally scheduled every 3–5 days. As this was a retrospective cohort, the actual timing and frequency of serial ultrasound scans were adjusted at the discretion of the attending neonatologist according to clinical status, hemorrhage severity, and progressive ventricular findings; no unified prospective scanning protocol was applied for all participants. Ultrasound examinations were conducted using a Mindray M9 ultrasound system equipped with a 5–10 MHz transducer. Standard coronal and sagittal images were obtained for evaluation of intracranial structures.

Three ventricular linear measurements were recorded on standardized ultrasound planes: anterior horn width (AHW, measured at the level of the foramen of Monro on coronal plane), ventricular index (Levene index), and occipital-horn/posterior-horn depth (6, 26, 27). Only anterior-horn width was used to stratify the severity of imaging-defined post-hemorrhagic ventricular dilatation (PHVD). Ventricular index and occipital-horn depth were extracted from ultrasound reports as supplementary descriptive clinical observational variables. These two metrics were not incorporated into PHVD severity grading and were not used for statistical outcome analyses.

Fixed AHW-based cut-offs for imaging-defined PHVD severity were adopted from published neonatal neurosonography observational cohorts (6, 26, 28): mild PHVD, 5–7 mm; moderate PHVD, 8–10 mm; severe PHVD, >10 mm. These thresholds serve as descriptive imaging-stratification cut-offs and are not formal clinical intervention thresholds. Gestational-age-specific centile reference curves for ventricular dimensions were not implemented in the current retrospective analysis, and absolute-millimetre cut-offs were applied across all subjects. For each infant, the maximum PHVD severity grade observed during follow-up was used for subsequent analysis.

In the present study, the primary imaging outcome was imaging-defined post-hemorrhagic ventricular dilatation (PHVD), defined as ventricular enlargement demonstrated on cranial ultrasound following IVH/CPH, independent of clinical signs of raised intracranial pressure or whether patients received CSF-related intervention. Clinically-diagnosed hydrocephalus, which requires additional clinical evidence including accelerated head-circumference growth, bulging anterior fontanelle, suture diastasis or confirmed raised intracranial pressure, was not adopted as an outcome metric. Hydrocephalus requiring intervention was analysed as a separate treatment-related exposure variable for CSF-drainage or neurosurgical procedures, rather than a diagnostic category of ventricular dilatation. In the present cohort, maximal imaging-defined post-hemorrhagic ventricular dilatation (PHVD) was mostly identified on the initial ultrasound examination at the time of hemorrhagic brain injury diagnosis.

### MRI evaluation

Brain MRI was successfully acquired in 68 of 109 enrolled neonates; 29 infants died before MRI could be arranged, and the remaining 12 infants were lost to follow-up or did not complete MRI for clinical reasons. Thus, the MRI subgroup consisted exclusively of clinically stable survivors and is subject to selection and survivorship bias; all MRI analyses in this study are purely descriptive, and formal diagnostic-accuracy comparison against cranial ultrasound was not performed.

MRI examinations were performed on a 1.5-T clinical magnetic resonance scanner. Standard sequences included T1-weighted, T2-weighted, FLAIR, DWI, and SWI (susceptibility-weighted imaging). The median interval between initial bedside cranial ultrasound and MRI was 22 days (interquartile range 13–38 days). Median post-natal age at MRI was 28 days (IQR 16–44 days). No corrected-age adjustment was applied because all scans were performed during the neonatal and early post-neonatal period. Two experienced paediatric neuroradiologists independently interpreted all MRI datasets, and readers were blinded to prior cranial-ultrasound images and ultrasound reports. Discrepant interpretations between radiologists were resolved by consensus discussion. When cranial ultrasound and MRI provided discrepant anatomical findings, MRI was used to determine the final hemorrhage classification.

### Clinical characteristics, comorbidities, and treatment variables

Clinical information was collected from electronic medical records, including sex, gestational age, birth weight, singleton or multiple birth status, and relevant perinatal characteristics. Major neonatal complications were recorded, including neonatal respiratory distress syndrome (NRDS) or severe respiratory disease, sepsis, disseminated intravascular coagulation (DIC), necrotizing enterocolitis (NEC), multiple organ failure (MOF), and congenital heart disease (CHD). A composite clinical severity score was calculated according to the presence of these six major comorbidities, ranging from 0 to 6 points, with higher scores indicating a greater burden of systemic complications. Treatment-related variables included cerebrospinal fluid drainage through lumbar puncture, external ventricular drainage or other ventricular fluid diversion procedures, ventriculoperitoneal shunt placement, surgical evacuation of hematoma, and other neurosurgical interventions. These procedures were considered exposure variables in subsequent analyses.

### Follow-up and outcome assessment

The primary imaging outcome was the severity of post-hemorrhagic ventricular enlargement. Long-term clinical outcomes were classified into three categories: death, Neurodevelopmental abnormality, and favorable neurodevelopmental outcome. Neurodevelopmental abnormality was diagnosed by pediatric neurologists or rehabilitation specialists according to established clinical criteria, and motor impairment severity was assessed using the Gross Motor Function Classification System (GMFCS) (29). To explore the robustness of our findings, we also conducted a sensitivity analysis restricted to survivors, in which cerebral palsy (a severe subtype of neurodevelopmental impairment) was used as the dependent variable. Survivors underwent neurodevelopmental evaluation at a corrected age of 18–24 months. A favorable outcome was defined as a cognitive or motor composite score ≥85 on the Bayley Scales of Infant and Toddler Development, Third Edition (Bayley-III) (30, 31).

## Statistical analysis

Continuous variables were evaluated for distribution characteristics before analysis. Normally distributed variables were presented as mean ± standard deviation and compared among groups using one-way analysis of variance (ANOVA), followed by least significant difference (LSD) testing for pairwise comparisons when appropriate. Variables with non-normal distributions were expressed as median (interquartile range, IQR) and analyzed using the Kruskal–Wallis test. Categorical variables were summarized as number (percentage, %) and compared using Fisher's exact test. Collinearity pre-analysis before model fitting: Prior to constructing the primary multinomial logistic regression model, collinearity diagnostics were performed. Since SPSS multinomial logistic regression does not directly output variance inflation factor (VIF), an auxiliary ordinary-least-squares (OLS) linear regression with exactly the same set of candidate predictors was used to obtain tolerance and VIF values for multicollinearity screening. All VIF values were less than 5. No severe multicollinearity was detected, and all pre-specified predictors were retained in the final multinomial logistic regression model.

## Results

### Baseline clinical characteristics of the study cohort

To characterize the clinical profile and major neonatal complications of infants with hemorrhagic brain injury, we analyzed 109 neonates diagnosed with IVH, CPH, or both (screening process shown in Figure 1). The cohort included 82 males (75.2%) and 27 females (24.8%). Respiratory complications were common, with 64 infants (58.7%) presenting with NRDS or other severe respiratory disorders. Sepsis or septicemia was documented in 54 infants (49.5%), CHD in 48 (44.0%), DIC in 34 (31.2%), NEC in 25 (22.9%), and MOF in 18 (16.5%) (Table 1). These findings indicate that neonatal hemorrhagic brain injury frequently occurred in the setting of significant systemic complications, particularly respiratory disease and infection-related conditions.

**Table 1:** Major clinical complications in the study cohort.

Comorbidity	Number of cases	Percentage (%)
NRDS or severe respiratory disease	64	58.7
Sepsis or septicemia	54	49.5
Congenital heart disease (CHD)	48	44.0
Disseminated intravascular coagulation (DIC)	34	31.2
Necrotizing enterocolitis (NEC)	25	22.9
Multiple organ failure (MOF)	18	16.5

### Additional findings identified by MRI

To explore the additional information provided by MRI beyond cranial ultrasound findings, we analyzed the subgroup of infants who underwent MRI examination. Among the 109 enrolled infants, 68 (62.4%) completed cranial MRI, whereas 41 did not. Among those without MRI evaluation, 29 infants died before MRI could be performed, and 12 surviving infants were unable to complete MRI examination because of clinical limitations or other reasons.

MRI identified hemorrhagic lesions involving multiple anatomical regions. The most frequently involved sites included the ventricular system (57 cases), subarachnoid space (37 cases), parietal lobe (25 cases), temporal lobe (23 cases), and thalamo-basal ganglia region (20 cases). Additional MRI findings included deep hemorrhages involving the thalamus and basal ganglia, focal cerebellar hemorrhages, and hemorrhagic lesions not clearly characterized by ultrasound. Because individual infants could have hemorrhage affecting multiple anatomical sites, the sum of lesion counts exceeded the total number of patients (Table 2).

**Table 2:** Distribution of hemorrhagic lesions identified by imaging.

Hemorrhage location	Number of cases
Thalamo-basal ganglia region (including 8 cases with ventricular extension)	20
Extension into ventricles	11
Frontal lobe	17
Parietal lobe (including 2 cases with ventricular extension)	25
Temporal lobe (including 1 case with ventricular extension)	23
Occipital lobe	13
Cerebellar hemorrhage	13
Choroid plexus	9
Subarachnoid hemorrhage	37
Intraventricular hemorrhage	57

Within the MRI subgroup, ultrasound and MRI interpretations showed complete agreement in 47 cases and partial agreement in 18 cases regarding hemorrhage localization. Several discrepancies reflected the different imaging capabilities of the two modalities. For example, some basal ganglia hemorrhages with ventricular extension were classified as IVH on ultrasound without clear identification of the deep parenchymal component. In addition, several small cerebellar hemorrhages and focal lesions were detected by MRI but were not visualized on ultrasound. Conversely, some hemorrhagic findings identified by ultrasound were not clearly demonstrated on MRI. In this selected survivor subgroup, MRI identified additional hemorrhagic lesions including deep parenchymal hemorrhage, posterior-fossa hemorrhage, and small subarachnoid micro-hemorrhages that were not readily visualised by cranial ultrasound. These observations are descriptive only and cannot be used to compare diagnostic performance between modalities.

### Comparison of clinical and imaging characteristics according to hemorrhage pattern

To determine whether hemorrhage distribution was associated with differences in clinical characteristics, PHVD severity, and treatment requirements, infants were classified into three groups according to the primary hemorrhage pattern: isolated IVH (*n* = 44, 40.4%), isolated CPH (*n* = 41, 37.6%), and combined IVH with CPH (*n* = 24, 22.0%). Significant differences were observed among the three groups in gestational age at birth and length of hospital stay. The mean gestational age was 33.18 ± 0.76 weeks in the isolated IVH group, 34.32 ± 0.73 weeks in the isolated CPH group, and 36.17 ± 0.71 weeks in the combined hemorrhage group (*P* = 0.042). The isolated IVH group had a longer hospital stay compared with the other groups (34.16 ± 3.81 days vs. 22.24 ± 4.04 days and 22.13 ± 3.54 days, respectively; *P* = 0.043). No statistically significant differences were observed in birth weight, clinical composite score, sex distribution, or multiple gestation status.

PHVD severity differed significantly among hemorrhage patterns (*P* < 0.001). Absence of PHVD was more frequent among infants with isolated CPH (24/41, 58.5%) compared with isolated IVH (2/44, 4.5%) and combined IVH with CPH (3/24, 12.5%). Moderate-to-severe PHVD occurred in 27 infants (61.4%) with isolated IVH, 10 infants (24.4%) with isolated CPH, and 17 infants (70.8%) with combined hemorrhage. The proportion of infants receiving cerebrospinal fluid drainage or neurosurgical intervention did not significantly differ among the three groups (*P* = 0.155) (Table 3). Pair-wise comparisons among the three hemorrhage pattern groups are provided in Supplementary Table S1. These results indicate that hemorrhage distribution was strongly associated with ventricular dilation patterns, with IVH-containing hemorrhage patterns showing a substantially higher burden of moderate-to-severe PHVD, whereas isolated CPH was less frequently accompanied by ventricular enlargement.

**Table 3:** Comparison of clinical and imaging characteristics among different hemorrhage patterns.

Variable	Isolated IVH (*n* = 44)	Isolated CPH (*n* = 41)	Combined IVH + CPH (*n* = 24)	*P* value
Gestational age at birth (weeks)	33.18 ± 0.76	34.32 ± 0.73	36.17 ± 0.71	**0**.**042**
Clinical composite score	1.75 ± 0.24	2.63 ± 0.29	2.21 ± 0.40	0.076
Birth weight (g)	2,208 ± 143	2,404 ± 138	2,733 ± 140	0.065
Hospital stay (days)	34.16 ± 3.81	22.24 ± 4.04	22.13 ± 3.54	**0**.**043**
PHVD severity				**<0**.**001**
None	2 (4.5%)	24 (58.5%)	3 (12.5%)	
Mild	15 (34.1%)	7 (17.1%)	4 (16.7%)	
Moderate	12 (27.3%)	6 (14.6%)	8 (33.3%)	
Severe	15 (34.1%)	4 (9.8%)	9 (37.5%)	
Drainage/surgical intervention	19 (43.2%)	10 (24.4%)	10 (41.7%)	0.155

Bold values indicate statistical significance (*P* < 0. 05).

### Follow-up outcomes and univariate analysis

To evaluate the relationship between hemorrhage characteristics, clinical variables, and neurodevelopmental outcomes, we analyzed mortality, Neurodevelopmental abnormality, and favorable neurodevelopmental outcomes during follow-up. Among the 109 infants included in the study, 41 (37.6%) died, 21 (19.3%) were diagnosed with Neurodevelopmental abnormality, and 47 (43.1%) achieved favorable neurodevelopmental outcomes at follow-up. The associations between clinical and imaging variables and follow-up outcomes are summarized in Table 4. Pairwise effect estimates (mean differences), adjusted *P*-values, and 95% confidence intervals obtained from Tukey–HSD *post-hoc* multiple comparisons are provided in the Supplementary Table S2.

**Table 4:** Association between clinical/imaging variables and follow-up outcomes.

Variable	Death s(*n* = 41)	Neurodevelopmental abnormality (*n* = 21)	Favorable outcome (*n* = 47)	*P* value
Gestational age at birth (weeks)	33.48 ± 0.68	36.30 ± 0.95	35.54 ± 0.67	**0**.**001**
Clinical composite score	3.58 ± 0.26	1.47 ± 0.22	1.32 ± 0.21	**<0**.**001**
Birth weight (g)	2,210 ± 139	2,545 ± 185	2,493 ± 131	0.230
PHVD severity				0.791
None	10	5	14	
Mild	8	5	13	
Moderate	9	6	11	
Severe	14	5	9	
Hemorrhage pattern				0.693
Isolated IVH	13	10	21	
Isolated CPH	17	7	17	
Combined IVH and CPH	11	4	9	
Drainage/surgical intervention				**0**.**015**
Yes	10	5	24	
No	31	16	23	

Pair-wise effect estimates, adjusted P-values and 95% confidence intervals from Tukey-HSD *post-hoc* comparisons are provided in the Supplementary Table S2.

Bold values indicate statistical significance (*P* < 0. 05).

According to hemorrhage pattern, the isolated IVH group included 13 deaths, 10 cases of Neurodevelopmental abnormality, and 21 infants with favorable outcomes. The isolated CPH group included 17 deaths, 7 cases of Neurodevelopmental abnormality, and 17 infants with favorable outcomes. The combined IVH and CPH group included 11 deaths, 4 cases of Neurodevelopmental abnormality, and 9 infants with favorable outcomes. The distribution of clinical outcomes did not differ significantly among the three hemorrhage groups (*P* = 0.693).

PHVD severity was also not significantly associated with follow-up outcome categories (*P* = 0.791). Mortality was observed across all PHVD grades, including infants without PHVD, mild PHVD, moderate PHVD, and severe PHVD. Similarly, the presence of multiple hemorrhagic lesions was not significantly associated with outcome differences (*P* = 0.312).

Several clinical factors showed significant associations with outcome distribution. Infants who died had a lower gestational age at birth compared with survivors (33.48 ± 0.68 weeks vs. 36.30 ± 0.95 weeks in the Neurodevelopmental abnormality group and 35.54 ± 0.67 weeks in the favorable outcome group; *P* = 0.001). The clinical composite score was also higher among infants who died (3.58 ± 0.26) compared with those with Neurodevelopmental abnormality (1.47 ± 0.22) or favorable outcomes (1.32 ± 0.21; *P* < 0.001).

Hospitalization duration differed significantly among outcome groups (*P* < 0.001), with shorter hospitalization observed among infants who died. This finding likely reflects early mortality rather than a direct association between shorter hospitalization and outcome improvement. Birth weight, sex, and multiple gestation status were not significantly associated with outcome categories.

Among infants receiving cerebrospinal fluid drainage or neurosurgical intervention, 10 died, 5 developed Neurodevelopmental abnormality, and 24 achieved favorable outcomes. Among those without such interventions, 31 died, 16 developed Neurodevelopmental abnormality, and 23 had favorable outcomes. The distribution of outcomes differed between the two groups (*P* = 0.015). However, because these interventions were determined according to clinical severity and neurological status, this association should be interpreted cautiously. These findings suggest that overall clinical condition, particularly gestational maturity and systemic disease burden, may have a stronger association with survival outcomes than hemorrhage subtype or PHVD severity alone.

### Multivariate analysis of factors associated with follow-up outcomes

Before fitting the primary multinomial logistic regression model, collinearity pre-screening was completed. All predictors showed VIF < 5, and no severe multicollinearity was identified (Supplementary Table S4); therefore all pre-specified variables were entered into the model. To identify independent factors associated with mortality and Neurodevelopmental abnormality after adjusting for potential confounding variables, multinomial logistic regression analysis was performed using favorable neurodevelopmental outcome as the reference category. Variables included birth weight, gestational age at birth, sex, clinical composite score, PHVD severity, drainage or surgical intervention, hemorrhage multiplicity, and hemorrhage pattern. The results of the multinomial logistic regression analysis are presented in Table 5. Lower gestational age and higher clinical composite scores were independently associated with increased mortality risk. Each additional week of gestational age was associated with lower odds of death compared with a favorable outcome (OR = 0.583, 95% CI: 0.425–0.800, *P* < 0.001). Conversely, each one-point increase in the clinical composite score was associated with increased odds of mortality (OR = 2.625, 95% CI: 1.479–4.657, *P* < 0.001).

**Table 5:** Multinomial logistic regression analysis of factors associated with follow-up outcomes.

Variable	Neurodevelopmental abnormality vs. favorable outcome OR (95% CI)	*P* value	Death vs. favorable outcome OR (95% CI)	*P* value
Birth weight	0.999 (0.998–1.001)	0.508	1.001 (1.000–1.003)	0.153
Gestational age at birth	1.186 (0.883–1.594)	0.257	0.583 (0.425–0.800)	**<0**.**001**
Sex	2.163 (0.545–8.592)	0.273	0.281 (0.036–2.184)	0.225
Clinical composite score	0.953 (0.557–1.633)	0.862	2.625 (1.479–4.657)	**<0**.**001**
PHVD severity	1.695 (0.830–3.459)	0.147	1.969 (0.780–4.968)	0.152
Drainage/surgical intervention	0.115 (0.022–0.595)	**0**.**010**	1.643 (0.323–8.372)	0.550
Multiple vs. single hemorrhagic lesions	1.213 (0.337–4.365)	0.768	0.687 (0.126–3.728)	0.663
Hemorrhage pattern: isolated IVH	1.115 (0.237–5.260)	0.890	0.443 (0.061–3.204)	0.420
Hemorrhage pattern: isolated CPH	0.861 (0.122–6.063)	0.881	0.639 (0.083–4.910)	0.667

Bold values indicate statistical significance (*P* < 0. 05).

For Neurodevelopmental abnormality outcomes, receipt of cerebrospinal fluid drainage or neurosurgical intervention was associated with lower odds of Neurodevelopmental abnormality compared with a favorable outcome (OR = 0.115, 95% CI: 0.022–0.595, *P* = 0.010). However, because intervention decisions are influenced by disease severity, this association should not be interpreted as evidence of a preventive effect. No significant associations were observed between Neurodevelopmental abnormality or mortality and birth weight, sex, PHVD severity, number of hemorrhagic lesions, or hemorrhage pattern (all *P* > 0.05). We further performed a sensitivity analysis restricted to survivors, using cerebral palsy as the dependent variable to evaluate whether the identified risk factors remained consistent after excluding deaths. The results are shown in Table 6. These results indicate that systemic clinical severity and gestational maturity were the primary factors associated with mortality in this cohort, whereas hemorrhage distribution, PHVD severity, and lesion multiplicity alone were not independent predictors of long-term outcome after adjustment.

**Table 6:** Binary logistic regression sensitivity analysis: cerebral palsy versus favorable neurodevelopmental outcome.

Variables	B	S.E.	P	OR	95% CI
Imaging-defined PHVD	0.829	0.816	0.309	2.291	0.463–11.331
CSF-drainage or neurosurgical intervention	−1.638	0.687	0.017	0.194	0.051–0.747
Hemorrhage anatomical pattern	−0.253	0.634	0.69	0.777	0.224–2.692
Multiplicity of hemorrhagic foci	0.168	0.679	0.805	1.183	0.313–4.476
Birth weight (g)	0	0.001	0.606	1	0.998–1.001
Gestational age at birth (weeks)	0.14	0.126	0.267	1.15	0.898–1.473
Multiple gestation	0.738	0.93	0.427	2.093	0.338–12.948
Clinical composite score	−0.032	0.239	0.894	0.969	0.607–1.547

## Discussion

Herein, we found that the anatomical distribution of hemorrhage was strongly associated with the severity of post-hemorrhagic ventricular dilation. Infants with hemorrhage involving the ventricular system (either isolated IVH or combined IVH-CPH) demonstrated substantially higher rates of moderate-to-severe post-hemorrhagic ventricular dilation compared with those with isolated cerebral parenchymal hemorrhage. By contrast, moderate-to-severe ventricular dilation was uncommon among children with isolated parenchymal hemorrhage. These observations support ventricular involvement as a key contributor to disturbed cerebrospinal-fluid circulation following neonatal hemorrhagic brain injury.

Our finding that ventricular system involvement predisposes to more severe post-hemorrhagic ventricular dilation is consistent with classic neuropathological descriptions and several preterm IVH cohorts, which have established that intraventricular blood breakdown products drive inflammatory obstruction and impaired CSF resorption (26, 32). Intraventricular blood products can obstruct cerebrospinal-fluid pathways, impair CSF resorption, and trigger inflammatory cascades that drive sustained ventricular enlargement. When hemorrhage concurrently affects ventricles and brain parenchyma, concomitant parenchymal tissue injury, local edema, and neuroinflammation may further exacerbate secondary ventricular dilation (33). Most existing literature mainly focuses on the Papile-graded intraventricular hemorrhage scale; fewer studies stratify patients according to anatomical patterns that combine ventricular and parenchymal bleeding (34). Traditional grading systems for intraventricular hemorrhage focus largely on IVH severity grades according to ventricular blood burden. Our results add to existing literature by suggesting that anatomical-pattern classification may offer supplementary information to identify infants at elevated risk of post-hemorrhagic ventricular dilation. Nevertheless, hematoma volume and serial, quantitative measures of progressive ventricular change were not available in this retrospective cohort; further work using standardized quantitative imaging metrics is therefore warranted.

Notably, after adjustment for clinical covariates, hemorrhage anatomical pattern showed no statistical association with mortality or Neurodevelopmental abnormality. This result appears to diverge from several previous observational studies, which reported that parenchymal extension of hemorrhage represented a powerful adverse prognostic marker for long-term neurodevelopmental outcomes (34). Potential explanations for this discrepancy include differences in study population, inclusion and exclusion criteria, case-mix of gestational ages, and variable sets of confounders incorporated into multivariable adjustment. Many earlier cohorts predominantly enrolled extremely preterm infants with classic germinal-matrix IVH, whereas our cohort included a broader spectrum of gestational ages and mixed parenchymal-ventricular bleeding phenotypes. Taken together, our findings indicate that anatomical hemorrhage patterns are informative for anticipating ventricular complications but cannot fully explain heterogeneous neurological outcomes. Prognosis after neonatal intracranial hemorrhage is multifactorial, shaped by gestational maturity, systemic disease burden, secondary brain insults, therapeutic management, and post-discharge developmental support (5). This reinforces the viewpoint from prior work that isolated imaging lesion patterns are insufficient to predict individual neurodevelopmental trajectories, and systemic clinical status should be weighted heavily in risk assessment (5). Conventional anatomical categorization also does not capture clinically relevant features such as hematoma volume, temporal evolution of injury, or concomitant white-matter injury (34).

In the present cohort, lower gestational age and higher exploratory comorbidity-count score were statistically associated with mortality. The protective effect of higher gestational maturity against neonatal death after intracranial hemorrhage has been repeatedly demonstrated in large population-based neonatal cohorts (35, 36). This is consistent with prior reports demonstrating that immature cerebral hemodynamic autoregulation and high systemic illness burden increase vulnerability to fatal neonatal outcomes (35, 36). By comparison, hemorrhage subtype and severity of post-hemorrhagic ventricular dilation showed no independent statistical association with mortality after multivariable adjustment. While severe hemorrhagic brain injury is intuitively linked to high mortality, our analysis suggests that once systemic comorbidity burden and gestational age are accounted for, hemorrhage anatomical pattern *per se* confers limited additional prognostic value for death, which resonates with findings from some recent single-center neonatal brain injury studies. This underscores that overall systemic clinical status should not be overlooked when risk-stratifying these infants, rather than relying exclusively on imaging findings.

The observed association between cerebrospinal-fluid-directed drainage or neurosurgical intervention and reduced odds of Neurodevelopmental abnormality among survivors must be interpreted with considerable caution. Prior literature remains divided regarding whether early CSF diversion improves long-term neurodevelopment in infants with post-hemorrhagic ventricular dilation; randomized controlled evidence is still lacking, and observational studies are heavily confounded by baseline illness severity and treatment selection (6, 9). Although timely relief of cerebrospinal-fluid obstruction could theoretically mitigate secondary mechanical brain injury and elevated intracranial pressure, clinical decisions to undertake these procedures are driven by baseline disease severity, neurological status, and perceived survival prospects. Consequently, the detected association likely reflects baseline patient-selection and indication bias rather than a direct protective therapeutic effect. Prospective studies with uniform pre-specified intervention thresholds are required to clarify optimal procedural timing and potential neurodevelopmental benefits. Furthermore, different procedures including lumbar puncture, external ventricular drainage, ventriculoperitoneal shunt and hematoma evacuation were pooled as one composite exposure variable in the primary regression model. These interventions differ substantially in clinical indication, operative risk and intended therapeutic goal. Detailed information on intervention subtype, case count, indication and timing is provided in Supplementary Table S3. Selection bias, survivorship bias and immortal-time bias cannot be fully excluded, because only infants who survived long enough and remained clinically stable could receive these invasive procedures. Therefore, the significant statistical association does not constitute evidence of a protective treatment effect.

Imaging analyses from the MRI-only survivor subgroup illustrate the complementary nature of cranial ultrasound and MRI for evaluating neonatal hemorrhagic brain injury. Numerous previous small-series neonatal neuroimaging studies have described additional hemorrhagic lesions detected by MRI that were missed by bedside ultrasound (7, 13). However, most such reports did not adequately account for survivorship bias because MRI could only be performed in clinically stable patients, a limitation explicitly emphasized in the present work. Bedside cranial ultrasound remains the first-line imaging modality for critically ill neonates owing to its portability, repeatability, and suitability for serial monitoring of ventricular dynamics, as described above. In this selected subgroup of clinically stable survivors, MRI yielded supplementary anatomical information and could delineate deep-seated, small-sized, or occult hemorrhagic lesions that were difficult to appreciate on ultrasound. Importantly, these data originate exclusively from stable survivors and cannot serve as evidence for superior diagnostic performance of MRI relative to ultrasound, consistent with recent imaging consensus statements for critically ill neonates. Serial cranial ultrasound should constitute the cornerstone of bedside surveillance, while MRI may be reserved for selected stable infants when additional anatomical detail is clinically indicated.

Several limitations of this single-center retrospective cohort need to be acknowledged when interpreting results. First, the sample size is moderate, and the number of cerebral-palsy endpoints was limited, raising concerns regarding statistical power especially for subgroup analyses. Second, hematoma volume, serial quantitative ventricular measurements, and granular markers of secondary brain injury were not systematically abstracted from historical charts. Third, our exploratory comorbidity-count score lacks external validation and assigns equal weight to heterogeneous neonatal complications; we could not reliably time-stamp whether comorbidities preceded or followed the index hemorrhagic event. Fourth, key perinatal confounders including mode of delivery, antenatal corticosteroid exposure, invasive ventilation, surfactant use, and thrombocytopenia could not be fully extracted for all patients due to incomplete archival documentation, leaving residual unmeasured confounding. Among these, only mode of delivery had complete records; however, it was not included in the multivariable model owing to the limited sample size and the need to avoid model overfitting. The remaining four variables had substantial missing data and were therefore not included in the adjusted analyses. Fifth, MRI assessments were restricted to clinically stable survivors; 29 infants died prior to MRI acquisition, creating survivorship and selection bias. All MRI-related analyses are purely descriptive, and no formal head-to-head diagnostic-accuracy comparison between ultrasound and MRI was performed. Sixth, precise timestamps for individual therapeutic lumbar-puncture procedures could not be reliably retrieved from medical records. Death represents a competing outcome for neurodevelopmental assessment; we have reported mortality for the full cohort and neurodevelopmental outcomes restricted to surviving children. Seventh, this study did not use conventional Papile IVH grading. As a result, we could not directly adjust for traditional IVH severity when analyzing the association between hemorrhage distribution and clinical outcomes. Residual confounding caused by overall hemorrhage severity may therefore be present. Finally, follow-up was limited to early-childhood neurodevelopment; longer-term functional outcomes remain to be characterized in future investigations.

## Conclusion

Our study demonstrates that hemorrhage distribution is closely associated with the severity of post-hemorrhagic ventricular dilatation, with ventricular involvement identifying infants at higher risk of moderate-to-severe ventricular enlargement. However, hemorrhage pattern alone was insufficient to predict mortality or Neurodevelopmental abnormality, whereas lower gestational age and greater systemic disease burden were independent factors associated with mortality. The relationship between cerebrospinal fluid drainage or neurosurgical intervention and neurological outcome requires further validation. Cranial ultrasound remains essential for bedside surveillance, while MRI provides complementary information regarding deep and occult brain injuries. A comprehensive assessment integrating hemorrhage pattern, ventricular progression, clinical severity, and individualized imaging evaluation may improve risk stratification and management of neonatal hemorrhagic brain injury.

## Data Availability

The raw data supporting the conclusions of this article will be made available by the authors, without undue reservation.
