# Supplementary material for: Hemorrhage distribution, imaging-defined post-hemorrhagic ventricular dilatation, and clinical outcomes in neonates with intraventricular and cerebral parenchymal hemorrhage: a single-center retrospective cohort study
Source: Front Pediatr. 2026 Oct 22;14:1965213. doi: 10.3389/fped.2026.1965213 (PMC13736738; doi:10.3389/fped.2026.1965213)
Supplement: Supplementary file 1 [file Table1.doc]

**Supplementary Table 1. Pair-wise comparisons using Tukey-HSD post-hoc test among three hemorrhage‑pattern groups**

| **Variable** | **Group comparison** | | **Mean difference (I‑J)** | **Std. Error** | **Adjusted P** | **95% CI** |
| --- | --- | --- | --- | --- | --- | --- |
| PHVD severity | Isolated IVH vs Isolated CPH | 1.153 | | 0.217 | <0.001 | 0.64 - 1.67 |
|  | Isolated IVH vs Combined IVH‑CPH | ‑0.049 | | 0.254 | 0.979 | ‑0.65 - 0.55 |
|  | Isolated CPH vs Combined IVH‑CPH | ‑1.202 | | 0.257 | <0.001 | ‑1.81 - ‑0.59 |
| Drainage/surgical intervention | Isolated IVH vs Isolated CPH | 0.188 | | 0.104 | 0.171 | ‑0.06 - 0.43 |
|  | Isolated IVH vs Combined IVH‑CPH | 0.015 | | 0.121 | 0.991 | ‑0.27 - 0.30 |
|  | Isolated CPH vs Combined IVH‑CPH | ‑0.173 | | 0.123 | 0.341 | ‑0.46 - 0.12 |
| Birth weight（g） | Isolated IVH vs Isolated CPH | ‑196.146 | | 189.54 | 0.557 | ‑646.70 - 254.41 |
|  | Isolated IVH vs Combined IVH‑CPH | ‑525.292 | | 221.581 | 0.051 | ‑1052.01 - 1.42 |
|  | Isolated CPH vs Combined IVH‑CPH | ‑329.145 | | 224.424 | 0.311 | ‑862.62 - 204.33 |
| Gestational age（weeks） | Isolated IVH vs Isolated CPH | ‑1.135 | | 0.999 | 0.494 | ‑3.51 - 1.24 |
|  | Isolated IVH vs Combined IVH‑CPH | ‑2.985 | | 1.168 | 0.032 | ‑5.76 - ‑0.21 |
|  | Isolated CPH vs Combined IVH‑CPH | ‑1.850 | | 1.183 | 0.266 | ‑4.66 - 0.96 |
| Clinical composite score | Isolated IVH vs Isolated CPH | ‑0.884 | | 0.385 | 0.061 | ‑1.80 - 0.03 |
|  | Isolated IVH vs Combined IVH‑CPH | ‑0.458 | | 0.45 | 0.567 | ‑1.53 - 0.61 |
|  | Isolated CPH vs Combined IVH‑CPH | 0.426 | | 0.456 | 0.62 | ‑0.66 - 1.51 |

Mean difference (I‑J) derived from Tukey‑HSD post‑hoc test. Mean difference > 0 indicates higher value in group I. 95% CI, 95% confidence interval. All P‑values were adjusted for multiple comparisons.
