# Supplementary material for: Hemorrhage distribution, imaging-defined post-hemorrhagic ventricular dilatation, and clinical outcomes in neonates with intraventricular and cerebral parenchymal hemorrhage: a single-center retrospective cohort study
Source: Front Pediatr. 2026 Oct 22;14:1965213. doi: 10.3389/fped.2026.1965213 (PMC13736738; doi:10.3389/fped.2026.1965213)
Supplement: Supplementary file 2 [file Table2.doc]

# Supplementary Table 2. Pair‑wise group comparisons using Tukey‑HSD post‑hoc test

| **Variable** | **Group comparison** | **Mean difference (I‑J)** | **Std. Error** | **Adjusted P‑value** | **95% CI** |
| --- | --- | --- | --- | --- | --- |
| PHVD severity | Favorable outcome vs Neurodevelopmental abnormality | ‑0.205 | 0.3 | 0.775 | ‑0.92 - 0.51 |
|  | Favorable outcome vs Death | ‑0.339 | 0.244 | 0.351 | ‑0.92 - 0.24 |
|  | Neurodevelopmental abnormality vs Death | ‑0.135 | 0.307 | 0.899 | ‑0.86 - 0.59 |
| Drainage/surgical intervention | Favorable outcome vs Neurodevelopmental abnormality | 0.273 | 0.123 | 0.072 | ‑0.02 - 0.56 |
|  | Favorable outcome vs Death | 0.267 | 0.1 | 0.023 | 0.03 - 0.50 |
|  | Neurodevelopmental abnormality vs Death | ‑0.006 | 0.125 | 0.999 | ‑0.30 - 0.29 |
| Hemorrhage pattern | Favorable outcome vs Neurodevelopmental abnormality | 0.03 | 0.203 | 0.988 | ‑0.45 - 0.51 |
|  | Favorable outcome vs Death | ‑0.207 | 0.165 | 0.425 | ‑0.60 - 0.19 |
|  | Neurodevelopmental abnormality vs Death | ‑0.237 | 0.207 | 0.489 | ‑0.73 - 0.26 |
| Birth weight（ g） | Favorable outcome vs Neurodevelopmental abnormality | ‑52.139 | 231.962 | 0.973 | ‑603.53 - 499.26 |
|  | Favorable outcome vs Death | 282.79 | 188.852 | 0.296 | ‑166.13 - 731.71 |
|  | Neurodevelopmental abnormality vs Death | 334.929 | 237.146 | 0.338 | ‑228.79 - 898.65 |
| Gestational age at birth( weeks) | Favorable outcome vs Neurodevelopmental abnormality | ‑0.723 | 1.169 | 0.81 | ‑3.50 - 2.06 |
|  | Favorable outcome vs Death | 3.057 | 0.952 | 0.005 | 0.79 - 5.32 |
|  | Neurodevelopmental abnormality vs Death | 3.78 | 1.195 | 0.006 | 0.94 - 6.62 |
| Clinical composite score | Favorable outcome vs Neurodevelopmental abnormality | ‑0.109 | 0.383 | 0.956 | ‑1.02 - 0.80 |
|  | Favorable outcome vs Death | ‑2.242 | 0.312 | <0.001 | ‑2.98 - ‑1.50 |
|  | Neurodevelopmental abnormality vs Death | ‑2.132 | 0.391 | <0.001 | ‑3.06 - ‑1.20 |
