# Supplementary material for: Hemorrhage distribution, imaging-defined post-hemorrhagic ventricular dilatation, and clinical outcomes in neonates with intraventricular and cerebral parenchymal hemorrhage: a single-center retrospective cohort study
Source: Front Pediatr. 2026 Oct 22;14:1965213. doi: 10.3389/fped.2026.1965213 (PMC13736738; doi:10.3389/fped.2026.1965213)
Supplement: Supplementary file 3 [file Table3.doc]

**Supplementary Table 3. Types, case numbers, clinical indications and timing of cerebrospinal fluid‑related drainage and neurosurgical interventions**

| **Intervention type** | **No. of cases** | **Clinical indication** | **Timing (days after admission)** |
| --- | --- | --- | --- |
| Ommaya reservoir / external ventricular drainage | 8 | Basal ganglia hemorrhage breaking into ventricle, intraventricular hematoma, intracranial infection, hemorrhagic cerebral infarction, obstructive ventricular dilatation | Day 3‑30 |
| Ventriculoperitoneal shunt | 1 | Severe refractory post‑hemorrhagic ventricular dilatation | Day 30 |
| Craniotomy for hematoma evacuation | 5 | Intracranial hemorrhage | Day 7-19 |
| Repeated lumbar puncture for cerebrospinal fluid drainage | 30 | Post‑hemorrhagic ventricular dilatation | Day 1‑17 |
