# Supplementary material for: Hemorrhage distribution, imaging-defined post-hemorrhagic ventricular dilatation, and clinical outcomes in neonates with intraventricular and cerebral parenchymal hemorrhage: a single-center retrospective cohort study
Source: Front Pediatr. 2026 Oct 22;14:1965213. doi: 10.3389/fped.2026.1965213 (PMC13736738; doi:10.3389/fped.2026.1965213)
Supplement: Supplementary file 4 [file Table4.doc]

**Supplementary Table 4. Collinearity diagnostics for predictors entered into the primary multinomial logistic regression model**

| **Variables** | **Unstandardized coefficient B** | **S.E. of B** | **Standardized coefficient Beta** | **t** | **P‑value (Sig.)** | **Tolerance** | **VIF** |
| --- | --- | --- | --- | --- | --- | --- | --- |
| Constant | 0.381 | 0.743 | — | 0.513 | 0.61 | — | — |
| Imaging‑defined PHVD (0 = absent; 1 = any PHVD) | 0.1 | 0.157 | 0.097 | 0.638 | 0.526 | 0.65 | 1.54 |
| CSF‑drainage or neurosurgical intervention (0 = no; 1 = yes) | −0.288 | 0.124 | −0.308 | −2.318 | 0.024 | 0.857 | 1.167 |
| Hemorrhage anatomical pattern (reference: isolated IVH; 1 = isolated CPH) | −0.048 | 0.131 | −0.051 | −0.365 | 0.717 | 0.764 | 1.308 |
| Multiplicity of hemorrhagic foci (0 = single lesion; 1 = multiple lesions) | 0.065 | 0.144 | 0.066 | 0.451 | 0.654 | 0.707 | 1.414 |
| Birth weight (g) | −6.242E‑5 | 0 | −0.117 | −0.519 | 0.606 | 0.296 | 3.378 |
| Gestational age at birth (weeks) | 0.026 | 0.024 | 0.25 | 1.09 | 0.28 | 0.288 | 3.467 |
| Sex (0 = female; 1 = male) | 0.144 | 0.142 | 0.138 | 1.017 | 0.313 | 0.824 | 1.213 |
| Multiple gestation (0 = singleton; 1 = multiple gestation) | 0.101 | 0.192 | 0.07 | 0.523 | 0.603 | 0.841 | 1.189 |
| Exploratory comorbidity‑count score (0‑6 points) | −0.012 | 0.05 | −0.033 | −0.234 | 0.816 | 0.755 | 1.324 |
